# SILAC-based quantitative proteomics and microscopy analysis of cancer cells treated with the *N*-glycolyl GM3-specific anti-tumor antibody 14F7

**DOI:** 10.3389/fimmu.2022.994790

**Published:** 2022-11-09

**Authors:** Paula A. Bousquet, Dipankar Manna, Joe A. Sandvik, Magnus Ø. Arntzen, Ernesto Moreno, Kirsten Sandvig, Ute Krengel

**Affiliations:** ^1^ Department of Chemistry, University of Oslo, Oslo, Norway; ^2^ Department of Physics, University of Oslo, Oslo, Norway; ^3^ Department of Biosciences, University of Oslo, Oslo, Norway; ^4^ Facultad de Ciencias Básicas, Universidad de Medellín, Medellín, Colombia; ^5^ Department of Molecular Cell Biology, Institute for Cancer Research, The Norwegian Radium Hospital, Oslo, Norway; ^6^ Centre for Cancer Cell Reprogramming, Faculty of Medicine, University of Oslo, Oslo, Norway

**Keywords:** cytoskeleton, NeuGc GM3/Neu5Gc GM3, 14F7, SILAC, transcription factors, immunotherapy, ganglioside, glycosphingolipid

## Abstract

Cancer immunotherapy represents a promising approach to specifically target and treat cancer. The most common mechanisms by which monoclonal antibodies kill cells include antibody-dependent cell-mediated cytotoxicity, complement-dependent cytotoxicity and apoptosis, but also other mechanisms have been described. 14F7 is an antibody raised against the tumor-associated antigen NeuGc GM3, which was previously reported to kill cancer cells without inducing apoptotic pathways. The antibody was reported to induce giant membrane lesions in tumor cells, with apparent changes in the cytoskeleton. Here, we investigated the effect of humanized 14F7 on HeLa cells using stable isotope labeling with amino acids in cell culture (SILAC) in combination with LC-MS and live cell imaging. 14F7 did not kill the HeLa cells, however, it caused altered protein expression (MS data are available *via* ProteomeXchange with identifier PXD024320). Several cytoskeletal and nucleic-acid binding proteins were found to be strongly down-regulated in response to antibody treatment, suggesting how 14F7 may induce membrane lesions in cells that contain higher amounts of NeuGc GM3. The altered expression profile identified in this study thus contributes to an improved understanding of the unusual killing mechanism of 14F7.

## Introduction

The past few decades have seen much progress in the field of cancer immunotherapy (1–3). Many monoclonal antibodies are in advanced clinical development, and several are already licensed for clinical use (4, 5). Most clinically interesting antibodies bind to immune or cancer cells, triggering cell death. Antibodies can kill cells by different mechanisms, the most common being antibody-dependent cell-mediated cytotoxicity (ADCC), complement-dependent cytotoxicity (CDC) and induction of apoptosis (6, 7). Less frequent types of killing mechanisms include Fc-independent induction of cytotoxicity (without inducing morphological changes; often observed in cell death linked to apoptosis) and non-apoptotic mechanisms, where membrane lesions are formed upon treatment with mAbs (8–14).

14F7 is a clinically promising monoclonal antibody raised against the ganglioside NeuGc GM3, which represents an attractive target for cancer immunotherapy since this glycolipid is absent from healthy adult human tissues (15), but present in several malignancies (16–26). 14F7 is an IgG1 antibody with high affinity for its antigen (in the low nanomolar range) (19, 27–29). This interaction has been characterized structurally (complex with the carbohydrate part of the glycolipid) (30, 31) and by mutation analysis (32). In mouse models, 14F7 showed strong anti-tumor effects (20, 33). In order to prevent a possible human anti-mouse antibody response, and thereby increase its potential for immunotherapy, the original murine 14F7 mAb was humanized (14F7hT) (34). The cytotoxic properties of 14F7 were retained in the humanized variant, and no difference compared to the murine antibody was observed, however, 14F7hT also gained the ability to induce cell death by ADCC (35). While recent studies have found that anti-ganglioside antibodies of different IgG subclasses are commonly found in pathological processes (36), high-affinity anti-carbohydrate antibodies are rare (37).

The mechanism by which 14F7 activates signaling leading to cell death remains poorly understood. Carr et al. showed that 14F7-induced cell death in murine cancer cells (P3X63-Ag8.653) was caused by a complement-independent mechanism (20). A similar finding was reported by Casadesús et al., who observed complement-independent cell killing for 14F7 and a 14F7 variant that recognizes both NeuGc and NeuAc GM3 (38). Roque-Navarro et al. found in another murine tumor cell line (L1210) that 14F7 induced cell swelling and giant membrane lesions, but not the typical phenomena of apoptosis (DNA fragmentation, caspase activation or Fas mediation), suggesting a novel oncosis-like cell death mechanism (39–41). Several other antibodies with pore-formation mechanisms have been described in the literature (8, 12, 42, 43). Both Roque-Navarro et al. and Dorvignit et al. found indications of cytoskeletal involvement in 14F7-mediated cell death, but the details of this mechanism remain unexplored and there are currently no indications as to which cytoskeletal proteins may be involved (39, 44).

**Figure 1:**
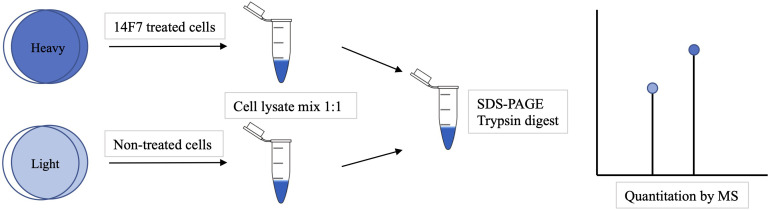
Overview of a SILAC experiment. First the cells were grown in 'light' or 'heavy' media. The 'heavy' cells were incubated with 14F7hT for 3 h before the cells were lysed and mixed. The proteins were separated by SDS-PAGE, trypsin-digested and identified by LC-MS analysis. The ratio of ‘heavy’ versus ‘light’ amino acids indicates which proteins were up- or down-regulated.

We have recently solved the crystal structure of the complex between 14F7 (a single-chain version) and the NeuGc GM3 trisacharide (31) and investigated how 14F7 recognizes NeuGc GM3 in a membrane-like environment (29). Here we seek to understand the effects that 14F7 induces in the cell, to gain a deeper understanding of the novel oncosis-like cell death mechanism induced by 14F7. To reveal differences in the expression profile between treated and untreated cells, we used stable isotope labeling with amino acids in cell culture (SILAC) in combination with LC-MS. Building on previous work (45, 46), we chose to work with HeLa cells. SILAC is a mass spectrometry (MS)–based quantitative method relying on the incorporation of ‘light’ and ‘heavy’ forms of amino acids (such as lysine and arginine) into proteins (**Figure 1**). It enables easy and comprehensive peptide identification by providing a defined number of labels per peptide (47). We identified twelve HeLa proteins that exhibited strongly altered expression after treatment with 14F7hT. Five of these proteins are related to the cytoskeleton and all of them were found to be downregulated in this investigation. No macroscopic changes were observed in the cells, however, this is likely due to the limited amount of NeuGc GM3 in the HeLa cell line.

## Materials and methods

### Cell culture

HeLa (ATCC: CCL-2) cells were incubated in Dulbecco’s Modified Eagle Medium (DMEM) supplemented with 10% (v/v) fetal bovine serum (FBS) to increase the amount of NeuGc GM3 as well as with 2 ml l-glutamine, 50 U/ml penicillin and 50 mg/ml streptomycin. The cells were kept at 5% CO_2_, 37°C between experiments and split when the confluence was approaching 80-90%. HeLa cells were seeded in 6, 24 or 96-well plates 24-72 h prior to experiments and incubated at 37°C in a 5% CO_2_ incubator. Experiments were performed using an unspecific secondary antibody as control.

### Incorporation of labeled amino acids and 14F7hT treatment of HeLa cells

For SILAC experiments, HeLa cells were cultured for at least five cell doublings in media either containing ^13^C- and ^15^N-labeled l-arginine (89990-Fisher) and ^13^C-labeled l-lysine (89988-Fisher) or media containing unlabeled l-arginine (89989-Fisher) and l-lysine (89987-Fisher) amino acids. The cells were treated with 14F7hT (25 μg/ml) for 3 h in 37°C, which was kindly provided by the Center of Molecular Immunology (CIM), Havana, Cuba.

### NanoLC-LTQ orbitrap mass spectrometry

HeLa cell lysates from each labeling, heavy and light, were mixed 1:1 and subjected to sodium dodecyl sulfate–polyacrylamide gel electrophoresis (SDS-PAGE). Each Coomassie G-250 stained SDS-PAGE gel lane was cut into 12 slices, destained at 37°C for 30 min, followed by reduction at 60°C for 10 min and alkylation for 1h in the dark. The samples, were in-gel digested at 37°C for 4h using 0.1 µg of trypsin in 25 µl of 50 mM ammonium bicarbonate, pH 7.8. After micropurification using µ-C18 ZipTips (Millipore, Oslo, Norway), the peptides were dried in a SpeedVac and dissolved in 10 µl 1% formic acid, 5% acetonitrile in water. Half of the volume was injected into an Ultimate 3000 nanoLC system (Dionex, Sunnyvale CA, USA) connected to a linear quadrupole ion trap-orbitrap (LTQ-Orbitrap XL) mass spectrometer (ThermoScientific, Bremen, Germany) equipped with a nanoelectrospray ion source. For liquid chromatography separation, an Acclaim PepMap 100 column (C18, 3 µm beads, 100 Å, 75 μm inner diameter) (Dionex, Sunnyvale CA, USA) capillary of 50 cm bed length was used. The flow rate was 0.3 μl/min, with a solvent gradient of 7% B to 35% B in 110 minutes. Solvent A was aqueous 0.1% formic acid, and solvent B aqueous 90% acetonitrile in 0.1% formic acid. The mass spectrometer was operated in the data-dependent mode to automatically switch between Orbitrap-MS and LTQ- MS/MS acquisition. Survey full scan MS spectra (from m/z 300 to 2,000) were acquired in the Orbitrap with the resolution R = 60,000 at m/z 400. The method used allowed the sequential isolation of up to the seven most intense ions for fragmentation on the linear ion trap using collision-induced dissociation (CID) at a target value of 10,000 charges. Target ions already selected for MS/MS were dynamically excluded for 60 sec. The lock mass option was enabled in MS mode for internal recalibration during the analysis. Other instrument parameters were set as previously described (48).

### Protein identification and quantification

Protein identification and quantification were performed with MaxQuant (49) (v.1.2.2.5) utilizing the Andromeda search engine (50). The tolerance level for matching the database was 6 ppm for MS1 and 20 ppm for MS/MS. Trypsin was used as digestion enzyme, and two missed cleavages were allowed. Carbamidomethylation of cysteines was used as fixed modification, whereas variable modifications included protein N-terminal acetylation, oxidation of methionines, deamination of asparagines and glutamines, and formation of pyro-glutamic acid at N-terminal glutamines. For estimation of the false discovery rate (FDR), which is the rate of falsely discovered proteins in our dataset, we included the reversed sequences into the database search. All hits to the reversed database could thus be regarded as false hits. By restricting the number of matches to this database to only 1% of total matches, we thus proceeded with an FDR of 1% to ensure reliable protein identification. For quantification, at least two quantification events were required per protein, and we further required the proteins to be quantified in at least 2 of 3 biological replicates. Normalized protein ratios H/L were reported by MaxQuant and used as is for analysis. A Student’s t-test was used to assess ratio significances.

### Bioinformatics analysis

Functional annotation was performed using DAVID Bioinformatics Resources version 6.7 (51, 52) available at http://david.abcc.ncifcrf.gov/, using whole genome (*Homo sapiens*) as background), and Panther (http://www.pantherdb.org).

### Measurement of cellular protein synthesis

HeLa cells were washed with leucine-free 4-(2-hydroxyethyl)-1-piperazineethanesulfonic acid (HEPES)-buffered medium and incubated with increasing concentrations of 14F7hT for 3 h or 18h at 37°C. Cells were then incubated with leucine-free HEPES-buffered medium complemented with 2 µCi/ml [^3^H] leucine (PerkinElmer) at 37°C for 20 min before proteins were precipitated with 5% (w/v) trichloroacetic acid (TCA) and washed once with the same solution (48). Finally, the proteins were dissolved in 0.1 M KOH and radioactively labeled leucine incorporation was quantified by ß-counting with Tri-Carb 2100TR^®^ Liquid Analyzer (Packard Bioscience). Three independent experiments were performed with biological duplicates.

### Measurement of cellular ATP level

Quantitation of the cellular ATP level was performed following the prescribed protocol by the commercially available CellTiter^®^-Glo 3D Cell Viability Assay kit (Promega). Briefly, HeLa cells (1x10^4^ cells/well, 96-well plate) were washed with 200 µl/well leucine-free HEPES medium. Thereafter, 50 µl fresh leucine-free medium was added to each well. 14F7hT was added to corresponding plates at increasing concentrations (25 ng/ml to 25 µg/ml). The plate was then incubated for 20 h at 37°C. After incubation, 50 µl CellTiter^®^-Glo was added to each well, followed by an incubation of 10 min in the dark at room temperature. The signal was measured using Syngene Chemi-Genious. Three independent experiments were performed with biological duplicates.

### Structural interference microscopy and live cell imaging

HeLa cells were cultured as described before and seeded on coverslips. The cells were washed in PBS and then fixed in a 4% (w/v) paraformaldehyde solution at room temperature (Alfa Aesar) for 15 min and permeabilized in 0.1% Triton X-100 in PBS for 2 min. The cells were incubated with the relevant primary antibodies diluted in blocking solution (10% PBS in FCS) for 1 h at room temperature or at 4°C overnight. The cells were again washed in PBS and incubated with blocking solution for 5 min. They were then incubated with secondary antibodies for 1 h. After the final washing step, the coverslips were mounted on ProLong Gold (Molecular Probes) supplemented with the nuclear staining reagent 4′,6-diamidino-2-phenylindole (DAPI) overnight at 37°C. Detailed analysis of single cells was either performed by confocal microscopy (Zeiss LSM 780) and analyzed with IMAGEJ software or super-resolution 3D SIM imaging performed on a DeltaVision OMX V4 system (Applied Precision) equipped with an Olympus 60X numerical aperture (NA) 1.42 object, cooled sCMOS camera and 405, 488 and 642 nm diode lasers, Z-stacks covering the whole cell were recorded with a Z-spacing of 125 nm. A total of 15 raw images (five phases, three rotations) per plane were collected and reconstructed by using SOFTWORX software (Applied Precision).

For live cell imaging, cells were seeded in 50 mm MatTek glass bottom dishes. Images were captured under controlled CO_2_ conditions at 37°C with a DeltaVision microscope (Applied Precision), equipped with a live cell Elite TruLight Illumination System and cooled Photometrics CoolSNAP HQ2 charge-coupled device (CCD) camera. Optical sections were acquired by using a 60X objective (Olympus, Plan Fluor, NA 1.42) and images were deconvolved by using SOFTWORX software (Applied Precision).

## Results and discussion

Using a quantitative proteomics approach and bioinformatics analysis, we compared the expression profile of 14F7hT-treated HeLa cells with control cells. To increase the amount of NeuGc GM3, we supplemented the media with 10% FBS. An overview of the experimental strategy for SILAC is depicted in **Figure 1**. HeLa cells were maintained in SILAC medium (containing ‘light’ or ‘heavy’ forms of the amino acids lysine and arginine). The cells grown in the ‘heavy’ medium were treated with the anti-tumor antibody 14F7hT, while the cells grown in the ‘light’ medium served as control in the experiment. Cell lysates from each labeling were mixed 1:1 and fractionized by SDS-PAGE. After in-gel digestion, protein identification and quantification, bioinformatics analysis was performed.

### SILAC and bioinformatic data analyses

**Figure 2:**
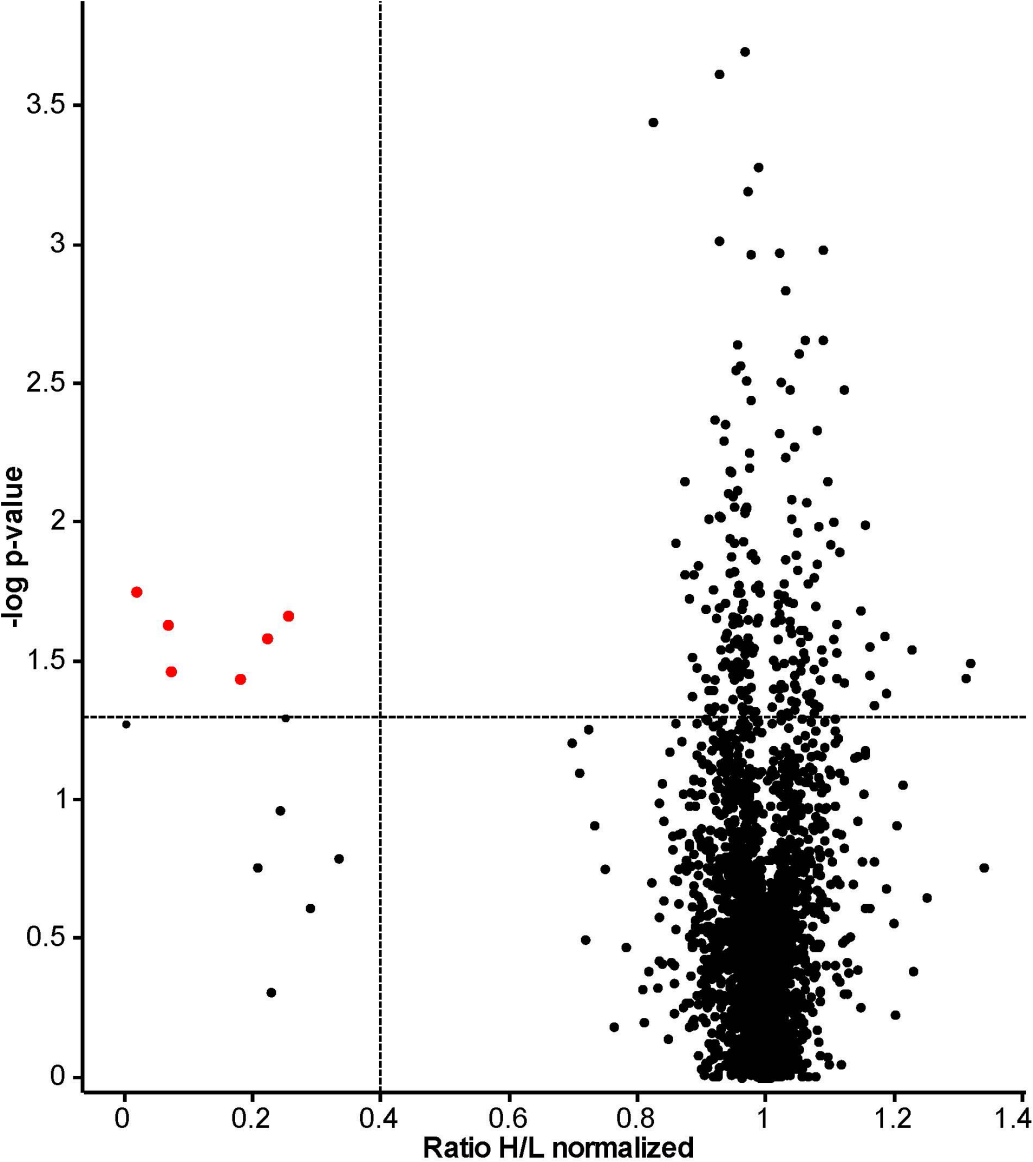
Volcano plot based on results obtained from 14F7hT-treated HeLa cells. This plot combines statistical significance, p-value (y-axis) with fold change (x-axis), to allow a quick visual overview over the interesting data. Points corresponding to the proteins with >2.5-fold altered expression levels and p < 0.05 are colored in red. The dotted lines represent the cut-off values (p < 0.05 = 1.3 at y-axis and 0.4, corresponding to 2.5-fold down-regulation, at x-axis).The discrepancy to **Table 1** results from the exclusion of proteins that did not fulfill our strict peptide criteria in the table, and exclusion of proteins without valid p-value from the plot. Excluded from **Table 1** were also two proteins identified as significant based on the p-value (labeled red in this plot): a glycosyltransferase (p = 0.024, H/L = 0.07, Protein ID: B7ZB85) and dermcidin, a secreted peptide with antimicrobial activity (p = 0.037, H/L = 0.18, Protein ID: P81605).

The proteomes of ‘heavy’ and ‘light’ HeLa cells, treated with 14F7hT and untreated, respectively, were compared by LC-MS. In total, 3685 proteins were identified. Two thirds of these proteins were quantified in at least two replicates and used for further analysis (**Table S1**). Following stringent criteria (p < 0.05, at least two peptides per protein in two of three replicates and a minimum fold-change of 2), four proteins were found to be significantly down-regulated (**Table 1**; note that in **Figure 2**, two of the “significant” hits had only few peptides). In addition, we identified one protein that was up-regulated 2.7-fold and seven proteins that were clearly down-regulated; however, with p-values >0.05 (or where p-values could not be obtained). Among these, one protein only marginally missed the target p-value (cystatin A, p = 0.051; 4-fold down-regulated), and two additional proteins had higher p-values, but at least two peptides in three replicates (kinesin-like protein KIF14, p = 0.490, H/L = 0.23; and F-actin-uncapping protein LRRC16A, p = 0.162, H/L = 0.33). For all of these proteins, p-values were <0.001 if calculated based on z-statistics instead. Adding these proteins to the number of strongly regulated proteins yields eleven down- and one up-regulated protein (**Table 1**). A volcano plot of all data points with valid p-value is shown in **Figure 2**. In addition, we screened the data for large differentials that may have been missed due to p-values >0.1 or less peptides identified in the replicates. The proteomics data have been deposited to the ProteomeXchange Consortium *via* the PRIDE partner repository, where they are freely accessible with the dataset identifier PXD024320 (53).

**Table 1:** Strongly regulated proteins.

Protein	Protein ID(Gene name)	H/Lratio	Function	Identified peptides	p-value^a^
**Down-regulated proteins**					
Dystrophin	P11532 (DMD)	0.02	cytoskeletal protein	4	0.018
Glucosamine/Glutamine-fructose-6-phosphate transaminase 2	O94808 (GFPT2)	0.07	controls the flux of glucose into the hexosamine pathway	5	0.035
CLIP-associating protein 2	B4DM73 (CLASP2)	0.07	cytoskeletal protein	7	n.a.
POTE ankyrin domain family, putative beta-actin-like protein 3	Q9BYX7 (POTEI)	0.13	ATP-binding cytoskeletal protein	6	n.a.
Brix domain containing 1/ribosome production factor 2 homolog	Q9H7B2 (RPF2)	0.20	associated with the nucleolus in an RNA-dependent manner	5	n.a.
Drug-sensitive protein 1/Gastric associated differentially-expressed protein YA61P	Q9NZ23 (YA61)	0.22	oxidoreductase activity	4	0.026
Kinesin-like protein KIF 14	Q15058 (KIF14)	0.23	cytoskeletal protein	5	0.490
Histidyl t-RNA synthetase, mitochondrial	P49590 (HARS2)	0.24	translation	7	n.a.
Cystatin A	P01040 (CSTA)	0.25	cytoskeletal protein	2	0.051
DERP12	Q8TE01 (DERP12)	0.26	oxidoreductase activity, acting on a sulfur group of donors	9	0.022
F-actin-uncapping leucine-rich repeat protein LRRC16A	Q5VZK9 (LRRC16A)	0.33	cytoskeletal protein	2	0.162
**Up-regulated proteins**					
Metastasis associated protein	Q13330 (MTA1)	2.70	identified in a screen for genes expressed in metastatic cells	2	n.a.

a p-values were based on Student’s t-test. Listed are proteins significantly regulated (p < 0.05 and >2-fold change, corresponding to normalized H/L ratio) upon 14F7hT mAb binding to HeLa cells. In addition, the list includes three proteins with higher p-values and five proteins, for which no valid t-test could be carried out (but which all had at least two peptides per protein in two of three replicates). The function was assigned using DAVID (51, 52).

n.a. (= not available, since there were not sufficient values for a valid t-test).

**Figure 3:**
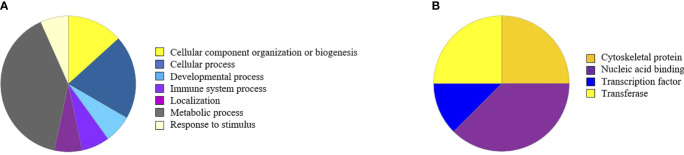
Regulated proteins visualized in pie diagrams according to biological process **(A)** or protein class **(B)**. Of note, evaluation with a newer version of PANTHER gave slightly different results (see **Figure S1**). However, while 14F7 treatment clearly affects metabolic and cellular metabolic processes, no particular metabolic pathways is singled out.

The bioinformatic tools DAVID (51, 52) and PANTHER (http://www.pantherdb.org) were used to categorize the regulated proteins (**Figure 3**).

19 transcription factors were identified as interacting partners of the 12 strongly regulated proteins listed in **Table 1**. These interactions were generated by DAVID protein-protein interaction analysis and are listed in **Table 2**. Three of the transcription factors interacting with the regulated proteins belong to the so-called homeobox genes. These genes express proteins that are spatially and temporally regulated during embryonic development (MEIS1B, HOXA3, TGIF). Several transcription factors (MEF2, GR, HSF2, EVI1, GATA and STAT) are also involved in cell development and growth. IRF and STAT are associated with interferon regulation and cell survival. Another transcription factor (JunB, ID: P17275) was directly down-regulated, by 1.4-fold, with 43 identified peptides (although slightly missing our criteria concerning p-value, with p = 0.062. This transcription factor is involved in regulating gene activity following primary growth factor response.

**Table 2:** Transcription factors interacting with the regulated proteins.

Transcription factor	Proteins interacting with transcription factor	Fold enrichment	p-value
TGIF	P01040, P11532, O94808, P49590, Q5VZK9, Q15058, B4DM73, Q13330	2.56	0.003
CDPCR1	P01040, P11532, P49590, Q5VZK9, Q15058, B4DM73, Q13330	2.45	0.015
MRF2	P01040, P11532, Q9H7B2, P49590, Q5VZK9, Q15058, B4DM73	1.83	0.066
HNF3B	P11532, Q9H7B2, O94808, P49590, Q5VZK9, B4DM73	1.98	0.092
GR	P11532, Q9H7B2, O94808, P49590, Q5VZK9, Q15058, B4DM73, Q13330	2.05	0.014
HSF2	P11532, Q9H7B2, O94808, P49590, Q5VZK9, B4DM73, Q13330	2.27	0.023
EVI1	P01040, P11532, Q9H7B2, O94808, P49590, Q5VZK9, Q15058, B4DM73, Q13330	1.41	0.062
GATA	P01040, P11532, O94808, P49590, Q5VZK9, Q15058, Q13330	2.11	0.033
IRF1	P11532, Q9H7B2, O94808, P49590, Q15058	2.73	0.059
STAT	P11532, O94808, P49590, Q5VZK9, Q15058, B4DM73	2.33	0.048
OCT	P11532, Q9H7B2, O94808, P49590, Q5VZ09, B4DM73, Q13330	2.04	0.039
LMO2COM	P01040, P11532, O94808, P49590, Q5VZK9, Q15058, B4DM73, Q13330	1.80	0.032
FOXJ2	P11532, Q9H7B2, O94808, P49590, Q5VZK9, Q15058, B4DM73, Q13330	1.59	0.068
MEIS1BHOXA9	P01040, P11532, Q9H7B2, P49590, Q5VZK9, Q15058, B4DM73	1.86	0.062
MEF2	P01040, P11532, Q9H7B2, O94808, P49590, Q5VZK9, Q15058, B4DM73, Q13330	1.58	0.026
SRY	P11532, P49590, Q5VZK9, Q15058, B4DM73, Q13330	1.94	0.098
HOXA3	P11532, P49590, Q5VZK9, Q15058, B4DM73, Q13330	1.95	0.097
FREAC2	P01040, P11532, O94808, P49590, Q5VZK9, Q15058, B4DM73	1.92	0.052
GATA1	P01040, P11532, Q9H7B2, O94808, P49590, Q5VZK9, Q15058, B4DM73, Q13330	1.43	0.057

### Proteins affecting the cytoskeleton

There are three major types of filaments in the cellular cytoskeleton, namely actin- and intermediate-filaments and microtubules, which all assemble from small building blocks. DAVID functional annotation analysis revealed five significantly down-regulated proteins belonging to the cluster of cytoskeletal proteins (**Table 1**). These proteins include cystatin A, CLIP-associating protein 2, leucine-rich repeat-containing protein 16A, Kinesin-like protein KIF 14 and dystrophin. Of additional interest is a member of the POTE ankyrin domain family, a pseudogene belonging to the actin family.

*Dystrophin (50-fold down-regulated).* A cytoskeletal protein present in a variety of tissues. It is involved in many biological processes and is associated with several disorders, in particular muscular and cardiac diseases (54–56). Surprisingly, this protein was almost completely absent in response to 14F7 treatment. Dystrophin has been connected to cell death, although the relationship is controversial. For example, the processes occurring in dystrophin-deficient muscle cells are linked to a pathological increase in intracellular Ca^2+^ concentration, which causes an increase in the volume of sarcoplasmic reticulum lumen (57–60). However, how the absence of dystrophin leads to increased cytosolic calcium levels is poorly understood, although damage to the membrane and defective calcium channels have been suggested as possible explanations (61–63). The cellular swelling observed in 14F7hT-treated cells may be partially explained by the down-regulation of dystrophin, if the same regulation occurs during 14F7hT-mediated cell death. Membrane damage is linked to the swelling phenotype of antibody-treated cells, and could be an explanation for an increased Ca^2+^ level. Ca^2+^ is stored and released by several organelles, in particular the acidic lysosomes (64), providing a link to the observed down-regulation of cystatin, sensitizing cells for lysosomal cell death.

*CLIP-associating protein 2 (14-fold down-regulated).* Regulation of the dynamic behavior of microtubules occurs through microtubule-associated proteins. Proteins that associate with the tips of microtubules are called +TIPs since they are ‘plus-end’ tracking proteins (65). The mechanisms used by +TIPs are not fully elucidated, but one of the significantly down-regulated proteins in response to 14F7hT mAb treatment, the cytoplasmic linker associated protein 2 (CLIP-associated protein 2, CLASP2) is a +TIP contributing to generate cellular asymmetry. A study using yeast two-hybrid analysis identified CLASP1 and CLASP2 as interaction partners to CLIPs (cytoplasmic linker proteins) (66). Interestingly, several proteins of this family (CLASP1, Cap-Gly domain of CLIP2 and CLIP1) were identified in the present work, but not considered significantly regulated according to our strict criteria.

In cells, the minus end of microtubules is localized deep in the microtubule-organizing center (MTOC), and microtubule bundles will grow out from the center. This will prevent dynamic instability at the minus end, but alternating between growth, pause and shrinkage will occur at the plus ends. When the microtubules grow towards the cell membrane, +TIPs, such as CLIP-associated protein 1, will ensure continuous growth until the microtubules reach the end, where shrinking can occur. This alteration from growth to shrinkage is termed ‘catastrophe’ (67–69). +TIPs thus function as anti-catastrophe factors, meaning that they prevent premature microtubules.

In studies using RNAi or antibodies targeting CLIP-associated protein 2, the formation of leading-edge-orientated microtubules was inhibited (66, 70). CLIP-associated protein was almost absent in the cells after 3 h of 14F7hT treatment. This can contribute to an inability of the microtubules to continuously grow, thus leading to morphological changes.

*POTE ankyrin domain family, putative beta-actin-like protein 3 (8-fold down-regulated).* Post-translational modifications of this pseudogene belonging to the actin family, such as oxidation and methylation, have (by similarity) been suggested to regulate polymerization of actin filament and actin-myosin processes like cleavage furrow ingression during cytokinesis, respectively. For the latter process, demethylation by a protein named alkylation repair homolog 5 (ALKBH) is required. This protein was identified, but not considered significantly regulated according to our criteria (fold change 0.88). Down-regulation of the POTE ankyrin protein caused by 14F7hT treatment could destabilize the processes of microtubule polymerization and cleavage furrow ingression.

*Kinesin-like protein KIF14 (4-fold down-regulated).* KIF14 is a motor protein playing an essential role in cytokinesis that has been associated with poor prognosis in breast cancer. It is localized in the nucleus during interphase (71) and associates with developing spindle poles and microtubules in mitotic cells, to then accumulate at the central spindle and midbody in the later stages of mitosis (72). The latter process is dependent on the presence of protein regulator of cytokinesis 1 (PRC1) and citron rho-interacting kinase (CIT). The expression levels of these proteins were altered, but not significantly. Carleton et al. showed that silencing of KIF14 generated a variety of mitotic phenotypes in HeLa cells, possibly linked to the efficacy of siRNA silencing (72). Using time-lapse microscopy, less efficacious silencing was shown to cause induction of distinct phenotypes, all resulting in acute apoptosis. However, a strong KIF14 silencing induced cytokinesis failure, resulting in multinucleated cells. This correlation between silencing efficacy and phenotypic outcome suggests that KIF14 alteration may disrupt different stages of the cell cycle, explaining the multitude of phenotypes reported (73–75). As KIF14 expression decreased significantly when cells were treated with 14F7hT, this may cause a phenotype change associated with cell fatality.

*Cystatin A (4-fold down-regulated).* Cystatin A (Stefin A) has been detected in higher levels in invasive tumors, where tumors positive for cystatin A were larger and exhibited an increased mitotic activity, suggesting a growth advantage for the cells (76). This protein was shown to be a potent inhibitor of exogenous proteases (77) and suggested to protect cytosolic and cytoskeleton proteins from degradation. High levels of cystatin A may be relevant for regulation of apoptosis by inhibiting cathepsin B, when initiated by the lysosomal cell death pathway. Cells lacking the closely related cystatin B (Stefin B) exhibit a higher sensitivity to lysosomal induced cell death (78). The significant down-regulation of cystatin A in 14F7hT-treated cells may sensitize the cells for lysosomal cell death as well as induce increased degradation of cytoskeletal proteins.

*F-actin-uncapping protein LRRC16A (3-fold down-regulated).* This *leucine-rich repeat* protein is also associated with actin polymerization. It was not clustered as cytoskeletal protein by DAVID, however, it decreases the affinity of capping proteins for actin ends by binding to the capping proteins (CAPZA2) with high affinity, thus inhibiting capping activity. Polymerization of actin filaments occurs *via* elongation at the end. By capping the ends, actin elongation terminates (79). Down-regulation of leucine-rich repeat-containing protein 16A, as observed in this study, may enhance the affinity for capping proteins to actin ends, hence leading to a termination of actin elongation.

Another cytoskeletal protein of potential interest is desmoplakin, a protein with a function in cell-to-cell adhesion. This protein was found to be down-regulated 1.4-fold (p = 0.080, 43 peptides; ID: P15924). Desmoplakin is involved in the organization of cadherin-plakoglobin complexes and in the anchoring of intermediate filaments to cell structures called desmosomes. In contrast, clathrin was slightly up-regulated (1.28-fold, p = n.a., 19 peptides; ID: P53675), which may suggest that 14F7 is taken up into cells by clathrin-dependent mechanisms.

### Other proteins up- or down-regulated

The only protein found to be significantly up-regulated (although not accessible to t-test statistics) was the metastasis-associated protein MTA1 (fold change 2.7; **Table 1**). MTAs belong to chromatin modifying proteins, functioning as integral parts of nucleosome remodeling and histone deacetylation (NuRD) complexes. MTA1 has been correlated with metastatic potential of carcinomas, but details of the process are poorly understood. However, it is known that MTA1 interacts with histone deacetylase 1 and 2 (HDAC1/2) (80), estrogen receptor alpha (81), CDK-activating kinase assembly factor MAT1 (MNAT1) (82) and tumor protein p53 (TP53) (83). Many cellular pathways are associated with MTA1, including cell fate programs. A possible explanation for MTA1 up-regulation upon antibody treatment is that it alters deacetylation of crucial target genes. Regarding the cell death mechanism, we noticed the down-regulation of TPX2 (fold change 0.82, p = 0.199, ID: Q96RR5; **Table S1**), which is involved in the assembly of microtubules during apoptosis, however, the effect was small, and contrary to what would be expected if cell death occurred by apoptosis. In contrast, a programmed cell-death protein (ID: Q9BRP1) was found to be almost 15-fold up-regulated, although with very weak criteria (p = n.a., 2 different peptides, but only one in two samples).

Two proteins with oxidoreductase activity, the drug-sensitive protein and the dermal papilla derived protein 12 (DERP12), were found to be down-regulated (fold change 0.22/0.26, p = 0.026/0.022) when HeLa cells were subjected to 14F7hT treatment (**Table 1**), whereas glutathione peroxidase was up-regulated 1.64-fold (p = n.a., ID: Q8TED1). Further studies will be required to suggest an explanation for the up- and down-regulated oxidoreductase activity.

Interestingly, one protein associated with carbohydrate biosynthesis, glucosamine/glutamine-fructose-6-phosphate aminotransferase 2, was found to be significantly down-regulated in our study (fold-change 0.07, *i.e.*, 14-fold, p = 0.035; **Table 1**), and an additional enzyme, glycosyltransferase-like protein, was found to be similarly down-regulated, although with less stringent criteria regarding the peptides (fold change 0.07, p-values = 0.024, ID: B7ZB85; **Table S1**). This is interesting since the target of 14F7, the NeuGc GM3 ganglioside, is a glycosphingolipid not normally present in human healthy cells, but found in the plasma membrane of several malignant cells (16–18). The synthesis of these gangliosides involves glycosyltransferases, which catalyze the attachment of carbohydrate residues to the hydrophobic ceramide part of the ganglioside. Decreased expression of these enzymes may be linked to down-regulation of the antibody target and/or associated with cellular metabolic processes. In contrast, we noticed that the catalytic subunit of dolichyl-oligosaccharyl transferase was slightly up-regulated, with good statistics (1.3-fold, p = 0.032, ID: P46977; **Table S1**).

Three other proteins that were found to be upregulated, by 4-, 5- and 18-fold, respectively, were a tyrosine phosphatase (ID: Q05209), an outer dense fiber protein (ID: Q5BJF6) and Hermansky-Pudlak syndrome protein, which is involved in the biogenesis of early melanosomes (ID: Q969F9) (84), however, all with rather poor statistics.

In this study, HeLa cells were analyzed, showing alteration in *e.g.* glycosylation, biosynthetic and primary metabolic processes, but other cell lines may have dissimilar glycolytic and lipid metabolic levels, affecting survival differently.

### 14F7hT neither inhibits protein synthesis nor changes cellular ATP level

**Figure 4:**
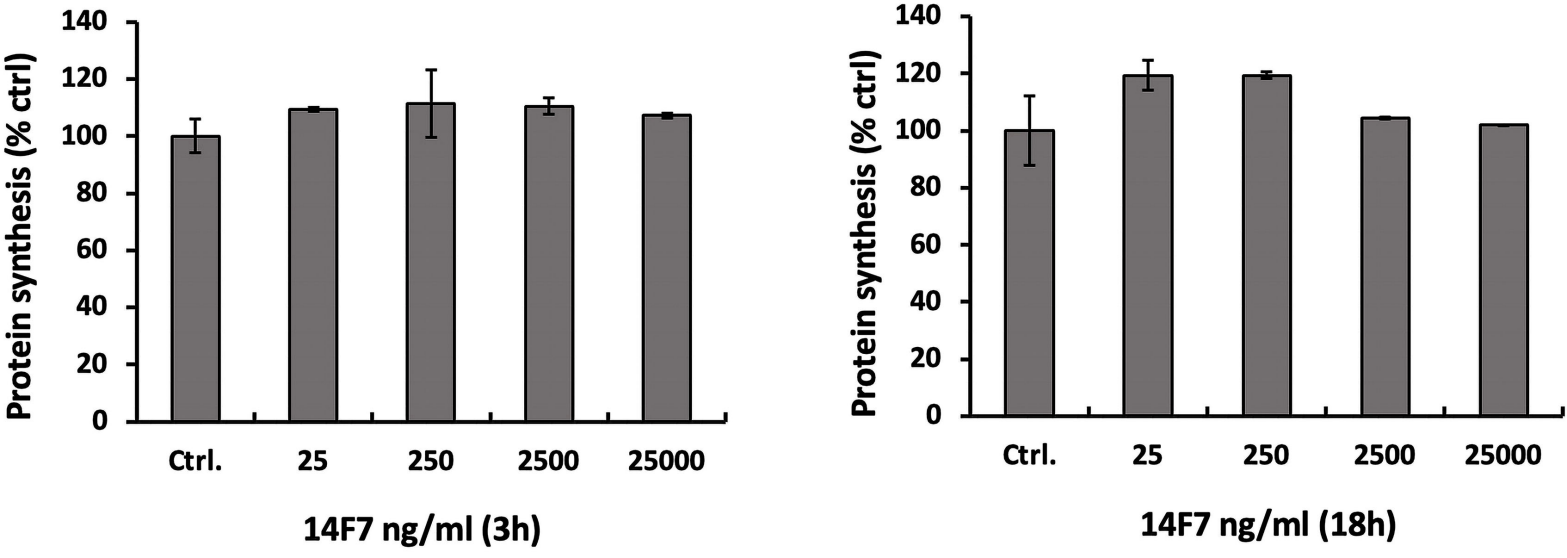
14F7hT treatment does not inhibit protein synthesis in HeLa cells. HeLa cells were incubated with varying concentrations (25 ng/ml to 25 µg/ml) of 14F7hT in serum-free medium for 3 h (left) or 18 h (right) at 37°C (n = 3). The level of protein synthesis was measured as described in the Methods section.

To investigate the toxic effect of 14F7hT more directly, we assessed protein synthesis of 14F7hT-treated cells. Measuring protein synthesis is a very sensitive method to study cell leakage. A 3h-treatment of HeLa cells with increasing concentrations of 14F7hT (25 ng/ml to 25 µg/ml) did not show any changes of cellular protein synthesis. In the four 14F7hT-treated samples, the total protein content remained unchanged compared to untreated cells (**Figure 4**). Even after 18 h, no changes were observed, indicating that 14F7hT treatment did not affect protein synthesis in the HeLa cells.

**Figure 5:**
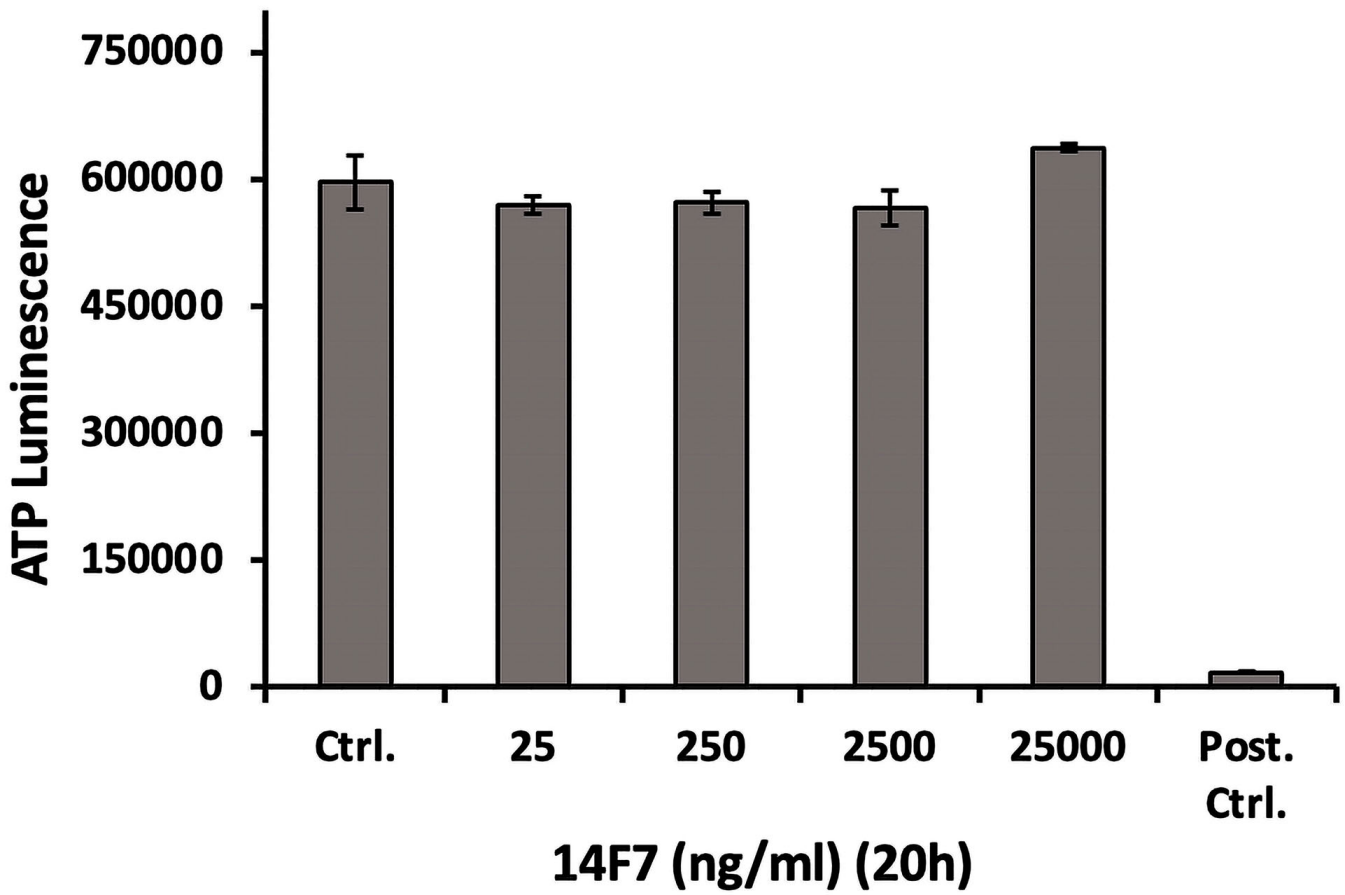
14F7hT mAb treatment does not affect the cellular ATP level. HeLa cells were treated with increasing concentrations (25 ng/ml to 25 µg/ml) of 14F7hT in leucine-free medium for 20 h at 37 °C (n = 3). The positive control contained a mixture of 10 mM NaN_3_ and 50 mM 2-deoxy glucose, and showed a strong decrease in cellular ATP, as expected.

We also investigated whether 14F7hT treatment would affect the cellular ATP level, since ATP depletion can lead to necrosis (85). To that end, we incubated HeLa cells with increasing concentrations of 14F7hT (25 ng/ml to 25 µg/ml) and subsequently measured the ATP level in the cells. The results showed no changes in the cellular ATP level 20 h after 14F7hT treatment (**Figure 5**), indicating that 14F7hT did not induce ATP leakage.

### 14F7hT does not disrupt the actin cytoskeleton or the microtubule network

**Figure 6:**
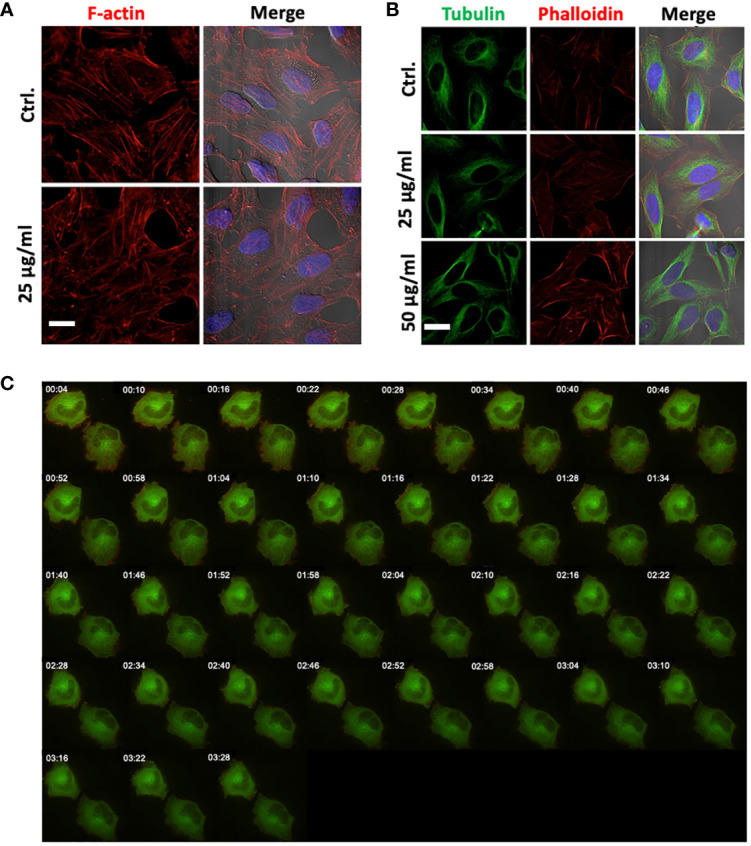
14F7hT mAb treatment does not lead to disruption of actin filaments or the tubulin network. HeLa cells were treated with either 25 µg/ml **(A, B)** or 50 µg/ml **(B)** 14F7hT and incubated for 6 h **(A)** or 15 h **(B)**, and thereafter stained for actin and tubulin, and analyzed by SIM microscopy (scale bar 7 µm). Fluorescence live cell imaging was performed to analyze the effects on actin (red) and tubulin (green) filament dynamics after treatment with 2 µg/ml 14F7hT over a period of 3.5 h **(C)**. 14F7hT treatment does not induce disruption or fragmentation of actin **(A)** or tubulin **(B)** filaments, nor does it have obvious effects on the dynamics of actin or tubulin filaments **(C)**. Representative pictures of three independent experiments are shown with >50 **(A, B)** and 10-20 visualized cells **(C)**, respectively.

Actin filaments and microtubules are important structural components of the cells, and interference with these components is associated with morphological changes or membrane disruptions. To evaluate the changes in cytoskeleton upon 14F7hT-treatment, we incubated HeLa cells with 14F7hT mAb, varying both 14F7 concentration and incubation times. The cells were stained for actin and tubulin to visualize the cytoskeleton with fluorescence microscopy (**Figures 6A, B**). For the analysis of filament dynamics, HeLa cells were transfected with RFP-actin and GFP-tubulin, to visualize the filaments by live cell microscopy after addition of 14F7hT (**Figure 6**). To our surprise, 14F7hT-treated cells did not display any morphological changes compared to non-treated cells. The HeLa cells contained an intact actin cytoskeleton and microtubule network with no obvious disruptions or fragmentations of these structural components even at the highest concentration (25 µg/ml) of 14F7hT and after long incubation times. Thus, the data clearly showed that the changes in expression profile revealed by SILAC are not manifested on the macroscopic level. The results obtained for the cells transfected with recombinant RFP-actin and GFP-tubulin, however, should be interpreted with caution since the expression levels of these proteins were artificially set with the transfection.

## Conclusion

Cancer immunotherapy is a growing research field. Several monoclonal antibodies are already applied in cancer therapy, and additional molecules are in the pipeline. These antibodies kill the malignant cells by different mechanisms, most commonly by ‘classical’ cell killing mechanisms, such as ADCC or CDC, but other mechanisms have also been suggested. 14F7hT has been reported to cause giant lesions in tumor cells and kill these cells by a non-apoptotic oncosis-like mechanism (39). Using a proteomics-based approach, we revealed 12 proteins that exhibited strongly altered expression upon 14F7hT binding to the target cells. Five of these are cytoskeletal proteins, affecting *e.g.* actin filament-based and microtubule-based processes. The HeLa cells studied in this work were not killed upon application of 14F7hT. Their NeuGc GM3 content is probably too low. Nevertheless, we suspect that the observed changes may represent early stages of cellular transformations that could be difficult to observe when the membrane lesions have formed and cells are dying. For example, we observed a slight down-regulation of TPX2 (1.2-fold), a protein involved in the assembly of microtubules during apoptosis, and 15-fold up-regulation of a programmed cell-death protein, although with poor statistics. A picture emerges that 14F7 treatment down-regulates proteins of the cytoskeleton and cell-cell-adhesion, and ultimately induces cell death. While further studies are required to verify the involvement of the identified proteins and the processes they inhibit or trigger, this work already considerably advances our current understanding of the 14F7 cell death mechanism, and identifies candidates for future therapies. An important question that remains unanswered is how this event of 14F7 binding to NeuGc GM3 on the cell membrane can connect with cell pathways that affect the expression of proteins related with the cytoskeleton.

## Data availability statement

Data are available *via* ProteomeXchange with identifier PXD024320.

## Author contributions

PB and UK conceived the study. PB carried out all SILAC-related experiments. MA processed the proteomics data. DM performed the microscopy studies, and analyzed protein inhibition and ATP levels, supervised by KS. UK served as main supervisor of PB and DM and coordinated the work. EM contributed to the discussion and manuscript revision. The first complete draft of the manuscript was written by PB and DM and revised in close collaboration with UK, with contributions from all authors. All authors contributed to the article and approved the submitted version.

## Funding

Work at UiO was funded by the University of Oslo (including the PhD position of PB). The PhD position of DM was funded by Norwegian loan funds. Work by EM at UdeMedellin was supported by Minciencias, Mineducación, Mincit and Icetex, through the Program NanoBioCáncer, grant no. FP44842-211-2018. Work in the lab of KS was supported by the Norwegian Cancer Society, grant no. 208239-2019.

## Acknowledgments

We wish to thank Sascha Pust for his strong support of the cell biology and microscopy experiments and co-supervision of DM, Nina F. J. Edin for advice regarding the SILAC experiments, Christian Köhler for assistance with data deposition into PRIDE, and Bernd Thiede for general support of the proteomics study. SP and BT also helped us to improve the manuscript. We further thank Erik O. Pettersen (Physics Department, University of Oslo) for access to his laboratory and support, and the Center of Molecular Immunology (CIM), Havana for providing us with 14F7hT mAb. The HeLa cell line was derived from Henrietta Lacks in 1951, who made significant contributions to biomedical research. We would like to thank her and her family members for this.

## Conflict of interest

EM was involved in developing 14F7 in the period 2000-2015 at the Center for Molecular Immunology in Havana, Cuba.

The remaining authors declare that the research was conducted in the absence of any commercial or financial relationships that could be construed as a potential conflict of interest.

## Publisher’s note

All claims expressed in this article are solely those of the authors and do not necessarily represent those of their affiliated organizations, or those of the publisher, the editors and the reviewers. Any product that may be evaluated in this article, or claim that may be made by its manufacturer, is not guaranteed or endorsed by the publisher.
