# Supplementary material for: SILAC-based quantitative proteomics and microscopy analysis of cancer cells treated with the N-glycolyl GM3-specific anti-tumor antibody 14F7
Source: Front Immunol. 2022 Nov 9;13:994790. doi: 10.3389/fimmu.2022.994790 (PMC9682173; doi:10.3389/fimmu.2022.994790)
Supplement: Supplementary file 1 [file DataSheet_1.pdf]

Supplementary information

SILAC-based quantitative proteomics and microscopy analysis of cancer cells treated with the *N*-glycolyl GM3-specific anti-tumor antibody 14F7

Paula A. Bousquet, Dipankar Manna, Joe A. Sandvik, Magus Ø. Arntzen, Ernesto Moreno, Kirsten Sandvig, Ute Krengel

Table S1. **Proteins analyzed with MaxQuant that qualified for Student's t-test** (.xlsx file).

Figure S1

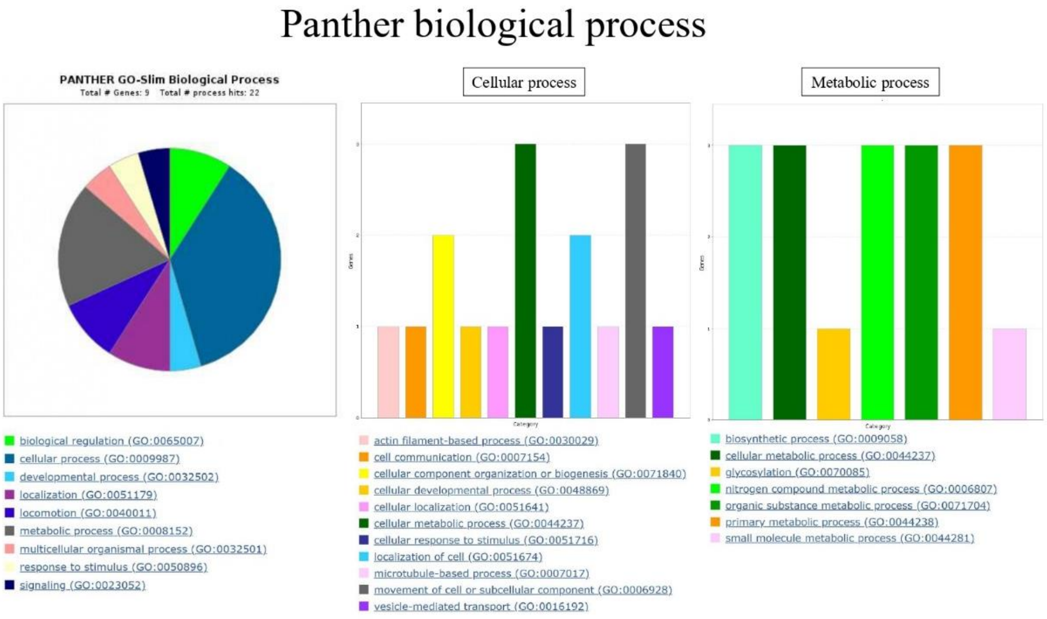

Figure S1. Regulated proteins visualized in pie diagrams according to biological process, according to the latest version of PANTHER (PANTHER.db; accessed on August 27th 2022).
